# Promoting Responsible Research and Innovation (RRI) During Brazilian Activities of Genomic and Epidemiological Surveillance of Arboviruses

**DOI:** 10.3389/fpubh.2021.693743

**Published:** 2021-07-01

**Authors:** Marta Giovanetti, Luiz Carlos Junior Alcantara, Alfredo Souza Dorea, Qesya Rodrigues Ferreira, Willian de Almeida Marques, Jose Junior Franca de Barros, Talita Emile Ribeiro Adelino, Stephane Tosta, Hegger Fritsch, Felipe Campos de Melo Iani, Maria Angélica Mares-Guia, Alvaro Salgado, Vagner Fonseca, Joilson Xavier, Elisson Nogueira Lopes, Gilson Carlos Soares, Maria Fernanda de Castro Amarante, Vasco Azevedo, Alícia Kruger, Gustavo Correa Matta, Laisa Liane Paineiras-Domingos, Claudia Colonnello, Ana Maria Bispo de Filippis, Carla Montesano, Vittorio Colizzi, Fernanda Khouri Barreto

**Affiliations:** ^1^Laboratório de Flavivírus, Instituto Oswaldo Cruz, Fundação Oswaldo Cruz, Rio de Janeiro, Brazil; ^2^Laboratório de Genética Celular e Molecular, Instituto de Ciencia Biologica (ICB), Universidade Federal de Minas Gerais, Belo Horizonte, Brazil; ^3^Instituição Beneficente Conceição Macedo, Salvador, Brazil; ^4^Instituto Multidisciplinar em Saúde, Universidade Federal da Bahia, Vitória da Conquista, Brazil; ^5^Laboratório de Virologia Molecular, Instituto Oswaldo Cruz, Fundação Oswaldo Cruz, Rio de Janeiro, Brazil; ^6^Laboratório Central de Saúde Pública, Fundação Ezequiel Dias, Belo Horizonte, Brazil; ^7^Laboratório de Desenvolvimento de Vacinas, Universidade de São Paulo, São Paulo, Brazil; ^8^Departamento Nacional de IST/AIDS/Hepatites Virais, Brasília, Brazil; ^9^Escola Nacional de Saúde Pública Sergio Arouca, Instituto Oswaldo Cruz, Fundação Oswaldo Cruz, Rio de Janeiro, Brazil; ^10^Departamento de Fisioterapia, Instituto de Ciências da Saúde, Universidade Federal da Bahia, Salvador, Brazil; ^11^Laboratorio di Scienze della Cittadinanza, Rome, Italy; ^12^Department of Biology, University of Rome Tor Vergata, Rome, Italy

**Keywords:** responsible research and innovation, Horizon 2020, public health, technological innovation, education

Scientific advances have been accompanied by great achievements and also great societal expectations with respect to research and innovation. In fact, the scientific scenario is reconfiguring itself. If research was previously limited to academy, now it is present in companies and has a direct relationship with economics and politics, playing an important role on social issues such as gender, accessibility, and opportunities (1).

In this sense, some world movements are emerging, as the Open Science and the Responsible Research and Innovation (RRI) (2, 3). These movements are transforming scientific practice, integrating and aligning the interests between science and technology, society and environment. The main aim is to expand the number of agents that think and execute these ideas, in addition to promoting greater accessibility to the results of scientific research and, consequently, performing a more popular science (4).

The European Commission defines RRI as “a process for better aligning research and innovation with the values, needs and expectations of society. It implies close cooperation between all stakeholders in various strands comprising science education, access to research results and the application of new knowledge in full compliance with gender and ethics considerations.” In practice, the RRI search for “science with and for society” and is guided by education as a promoter of scientific propagation (5). Actually, the information itself is not the same of a useful and critical knowledge. To become knowledge, it is necessary to disseminate the information, always based on open access. In this sense, continued training and the dissemination of technology are essential for a growing range of professionals to bring research and innovation to places where opportunities are usually scarce. In addition, the popularization of science and technology provides an increase in interest in issues that are usually away from routine, such as the genomic and epidemiological surveillance (1, 4).

Currently, the genomic and epidemiological surveillance of arboviruses seems to play a crucial role in Brazilian context. Seasonally outbreaks of dengue, chikungunya, zika, and yellow fever occurs in Brazil, disproportionately affecting the poorest population and overwhelmed the public health system (6). Coupling genomic diagnostics and epidemiology to innovative digital disease detection platforms allowed an open, global and digital viral pathogen surveillance system (7–10). In this context, considering viral pathogen surveillance in mind, real-time sequencing, bioinformatics tools and the combination of genomic and epidemiological data from viral infections can give essential information for understanding the past and the future of an epidemic, making possible to establish an effective surveillance framework on tracking the spread of infections to other geographic regions. These actions can be even more relevant if carried out in conjunction with educational actions, such as technology transfer and training courses.

In this sense, in order to assist the Brazilian Ministry of Health (BMoH), we carry out a real-time genomic monitoring of the arbovirus circulating and co-circulating in Brazil, during the 2016–2020 epidemics, applying the RRI concepts. These concepts involve five principal keys: (i) Gender; (ii) Open Access; (iii) Education; (iv) Public Engagement; and (v) Ethics. The following strategies were established: (i) a team based on achieving gender equality (the team comprised 24 people of which 12 male and 12 female); (ii) an open access database to make data available as soon as they were produced; (iii) technology transfer and capacity building for the health workers to track the spread of emerging viral pathogens; (iv) the publications of the obtained results in local and international open access journals to make available final data for the scientific community and the general population; and (v) all steps were carried out based on the application of fundamental ethical principles and legislation to scientific research (Figure 1).

**Figure 1:**
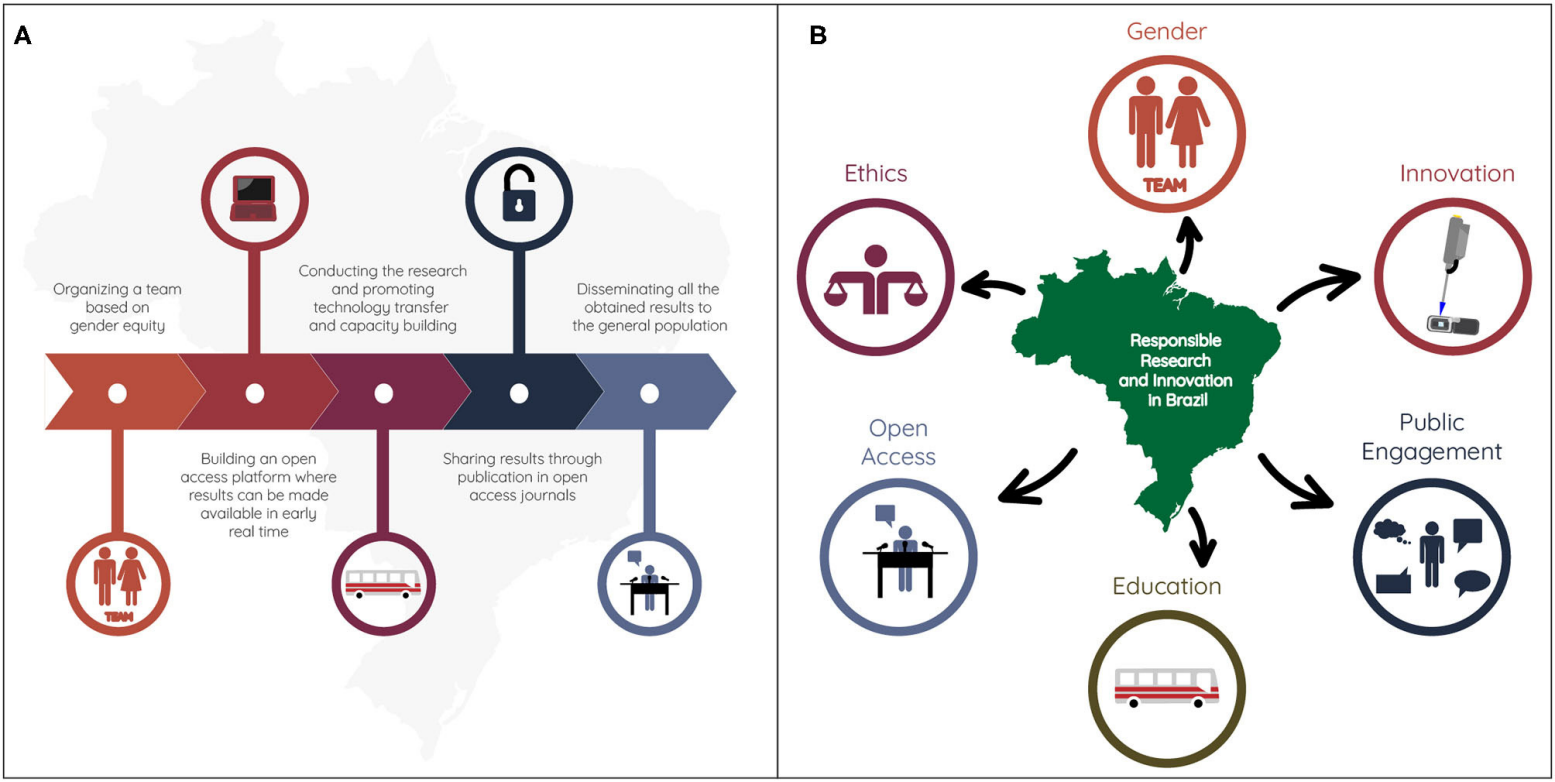
llustrative diagram representing the strategy used to implement RRI concepts during real-time genomic surveillance activities of circulating arboviruses carried out in Brazil. **(A)** Timeline showing the strategy steps. **(B)** The RRI principles applied during the research.

Our real-time genomic activities started in 2016 when Brazil and the Americas experienced the emerging of a new international concern the Zika virus infection. At that time in collaboration with National and International institutions we started our sequencing mission in Northeast Brazil. During this project, called ZiBRA (Zika in Brazil Real Time Analysis), we were capacitated on the use of the nanopore sequencing, to get more insight regarding the dispersion dynamics of this emerging viral treat in Brazil. Using a mobile lab in 15 working days we generate a substantial number of complete and near complete ZIKV genomes and all data were also shared in real time on a free online website platform (http://www.zibraproject.org) (11).

Considering the success obtained using this novel technology we decided to extend those activities to track the emergence and the re-emergence of other viral pathogens circulating in Brazil and Americas (https://www.zibra2project.org). For this purpose, we lunched the second phase of those real-time genomic activities, called ZIBRA-2 project, where we characterized all the arboviruses currently circulating and co-circulating, including ZIKV, chikungunya virus (CHIKV), dengue virus (DENV) and yellow fever virus (YFV).

We investigated the spread of the CHIKV—East/Central/South/African genotype (CHIKV-ECSA) in different Brazilian regions (North, Southeast and Southeast), as well as we followed the spread of ZIKV in the Amazon region (12–14). Between 2016 and 2018 we were also fully dedicated to track the re-emergence of YFV in Southeast, Brazil. In this context, we extended our real-time genomic surveillance activities to understand the origins of this outbreak firstly detected in the state of Minas Gerais and then follow the YFV spreading in other southeastern (Rio de Janeiro, Espírito Santo, and São Paulo) and Northeastern (Bahia). Those activities gave us the possibility to generate 200 complete and/or near YFV complete genome sequences from those regions, and highlighted the importance of genomics-based methods to infectious disease surveillance and control (9, 15). During these activities we also applied the RRI concepts, by targeting public health and higher-education institutions. We generate and analyze most of the data in real-time within training programs, and we provide a proof-of-concept of the unique opportunities that portable sequencing technologies offer for local capacity building.

In 2019, we take advantage of the experiences obtained and innovated upon such field surveillance initiatives by including real-time training sessions in the surveillance schedule under 2 formats: in the field, using a mobile nanopore sequencing laboratory inside a motorhome for 17 days in the Midwest, training personnel from local public health laboratories; and in the classroom, under a 2 week workshop (Belo Horizonte city, Southeast) attended by a large number of participants from public health laboratories from across Latin America.

The course had 62 students from 34 national and international institutions (age range of participants between 25 and 50 of which half of them were male and half female). In addition to post-graduate students, course participants included laboratory technicians and health practitioners in universities and laboratories from several institutions responsible for laboratory-based surveillance of emerging and reemerging diseases, such as the Central Public Health Laboratories of the Brazilian states from the BMoH's network and public health laboratories from Paraguay, Argentina, Panama, Chile, Mexico, Uruguay, Costa Rica, and Ecuador. In both formats, training included all surveillance tasks, from sequencing to computational analyses and research writing. Employing genomic surveillance in the field and in the classroom, we generated and analyzed 227 novel complete genome sequences of Dengue 1–2 (16).

In order to disseminate those experiences in our institution (FIOCRUZ), at the end of 2019 we promoted the meeting “Structural Transformations for Responsible Research and Innovation.” During this event we had the opportunity to discuss with researchers and graduate students ways to apply the RRI concepts, in addition to emphasizing the need of promote structural change in this field, in research institutions. Considering that Fiocruz is mainly involved in producing, disseminating and sharing knowledge and technologies, we had no problems in proposing measures to disseminate RRI concepts in the institution. People, researcher, students involved were really receptive to try to come together to make those structural changes happen. It is important to note that, part of the Fiocruz's mission, as stated before, is promoting the scientific dissemination to the general population. In this sense, we used to share in nearly real time all the obtained results with the general public, using social midia (video and television platforms) including: (i) YouTube, (ii) Twitter, and (iii) Brazilian open TV channels (more details can be found here: https://www.zibra2project.org/zibra-press/).

Overall, the surveillance outputs and training initiative carried out during those activities also served as a proof-of-concept for the utility of real-time portable sequencing for research and local capacity building in the genomic surveillance of emerging viruses. More recently, in March 2020, the Coronavirus Disease 2019 (Covid-19) pandemic, caused by the Severe Acute Respiratory Syndrome Coronavirus 2 (SARS-CoV-2) virus, was declared by The World Health Organization (WHO). In February, the Brazilian Ministry of Health confirmed the first case in the country and in April the LACEN from Minas Gerais state worked in collaboration with our team to sequence and analyze 40 complete SARS-CoV-2 genomes (17). This work can be seen as an example of the RRI impact on the interactions between science and society in Brazil and demonstrates the importance of the RRI concepts application during all research activities for social and health policy. A similar project of sequencing SARS-CoV2 genomes has been required by the Ministries of Health of the Republic of Chad and of the Republic of Cameroon (Central Africa). Two of the authors (M.G. e V.C.) are ready to move to Central Africa for capacity building, viral sequences and bioinformatics training in the context of the RRI approach.

## Conclusion

The data presented here reinforce the need for real-time and continued genomic surveillance strategies to better understand and prepare for the epidemic spread of emerging viral pathogens and fortify that this is only possible through the spread of science and technology combined with social and environmental awareness. This implies harm reduction and intensification of benefits for society, the economy and the environment, in addition to equipping the scientific community to act more quickly in times of crisis.

The participation in Structural Transformation to Attain Responsible BIOSciences (https://starbios2.eu), a European project that received funding from the Horizon 2020 and aims to implement the RRI approach in research institutions, provided us with the ideal environment to reflect our research projects, as well as facilitates the practical insertion of RRI into bioscience. We hope that this report can help the scientific community to promote structural changes in their research, in order to practice and disseminate the Responsible Research and Innovation.

## Author Contributions

MG, LA, and FB: conception and design and draft preparation. MG, LA, AD, QF, WM, JJ, TA, ST, HF, FI, MM-G, AS, VF, JX, EL, GS, MC, VA, AK, GC, LP-D, CC, AB, CM, VC, and FB: methodology. LA: resources. All authors contributed to the article and approved the submitted version.

## Conflict of Interest

The authors declare that the research was conducted in the absence of any commercial or financial relationships that could be construed as a potential conflict of interest.
